# Imaging tumor microenvironment heterogeneity in oral squamous cell carcinoma using IVIM DWI habitat analysis

**DOI:** 10.1186/s41747-026-00794-z

**Published:** 2026-09-09

**Authors:** Siyu Li, Xiaofeng Zheng, Yifeng Huang, Jiliang Ren, Yingwei Wu

**Affiliations:** 1 Department of Radiology, Shanghai Ninth People’s Hospital, Shanghai Jiao Tong University School of Medicine, Shanghai, China; 2 State Key Laboratory of Oral Diseases, National Center for Stomatology and National Clinical Research Center for Oral Diseases, Department of Oral Medical Imaging, West China Hospital of Stomatology, Sichuan University, Chengdu, China; 3 Department of Oral Pathology, Shanghai Ninth People’s Hospital, College of Stomatology, Shanghai Jiao Tong University School of Medicine, Shanghai, China; 4 Department of First Dental Clinic, Shanghai Ninth People’s Hospital, Shanghai Jiao Tong University School of Medicine, Shanghai Research, Institute of Stomatology, Shanghai, China

**Keywords:** Diffusion magnetic resonance imaging, Head and neck neoplasms, Lymphocytes (tumor-infiltrating), Squamous cell carcinoma of head and neck, Tumor microenvironment

## Abstract

**Objective:**

The tumor microenvironment (TME) of oral squamous cell carcinoma (OSCC) is spatially heterogeneous and critically influences prognosis. Conventional magnetic resonance imaging (MRI) diffusion-weighted imaging (DWI) metrics average heterogeneity and obscure relevant biological intratumoral patterns. Habitat imaging enables voxel-wise characterization of diffusion–perfusion heterogeneity.

**Materials and methods:**

Eighty-four patients with pathologically confirmed OSCC were prospectively enrolled and underwent preoperative multi-*b*-value intravoxel incoherent motion (IVIM)-DWI. A voxel-wise habitat imaging framework was applied based on diffusion-related (*D*_*t*_) and perfusion-related (*f*) parameters. Four habitats were defined using population-level *D*_*t*_ and *f* thresholds, corresponding to distinct combinations of diffusion and perfusion characteristics, to ensure standardized subregion definitions across patients. Tumor-stroma ratio (TSR), tumor-infiltrating lymphocytes (TILs), and cervical lymph node metastasis (CLNM) were assessed on histopathological sections. Habitat metrics, including subregional percentage, volume, and mean *D*_*t*_ and *f* values, were quantified and correlated with tumor-level pathological phenotypes. The predictive performance was evaluated.

**Results:**

High-TSR tumors exhibited higher *D*_*t*_ value within hypocellular habitats (subregions 3 and 4), which independently predicted a stroma-rich phenotype. High-TIL tumors demonstrated lower *D*_*t*_ in a cellular-dominant, high-perfusion habitat (subregion 2) and a reduced percentage of hypocellular, low-perfusion habitat (subregion 3), consistent with immune-enriched microenvironments. CLNM-positive tumors showed overall habitat expansion, with increased volume of a putative stroma-dominant habitat independently associated with metastasis. Habitat-based models outperformed whole-tumor apparent diffusion coefficient and *D*_*t*_ for predicting TSR (area under the receiver operating characteristic curve (AUC) = 0.791) and TILs (AUC = 0.782).

**Conclusion:**

IVIM-DWI-based habitat imaging provides a noninvasive approach for characterizing diffusion–perfusion heterogeneity in OSCC and may complement histopathological assessment for preoperative risk stratification.

**Key Points:**

- ***Question:*** Can IVIM-DWI-based habitat imaging noninvasively characterize tumor microenvironment heterogeneity and identify imaging features associated with stromal composition, immune infiltration, and nodal metastasis in OSCC?
- ***Findings:*** IVIM-DWI-based habitat imaging identified spatially distinct diffusion–perfusion habitats associated with TSR, TILs, and CLNM, with improved discrimination over conventional whole-tumor diffusion metrics.
- ***Relevance statement:*** IVIM-DWI-based habitat imaging provides clinically relevant, noninvasive biomarkers of TME features, improving preoperative risk stratification and supporting more precise, individualized therapeutic decisionmaking in OSCC.

**Graphical Abstract:**

**Figure:**
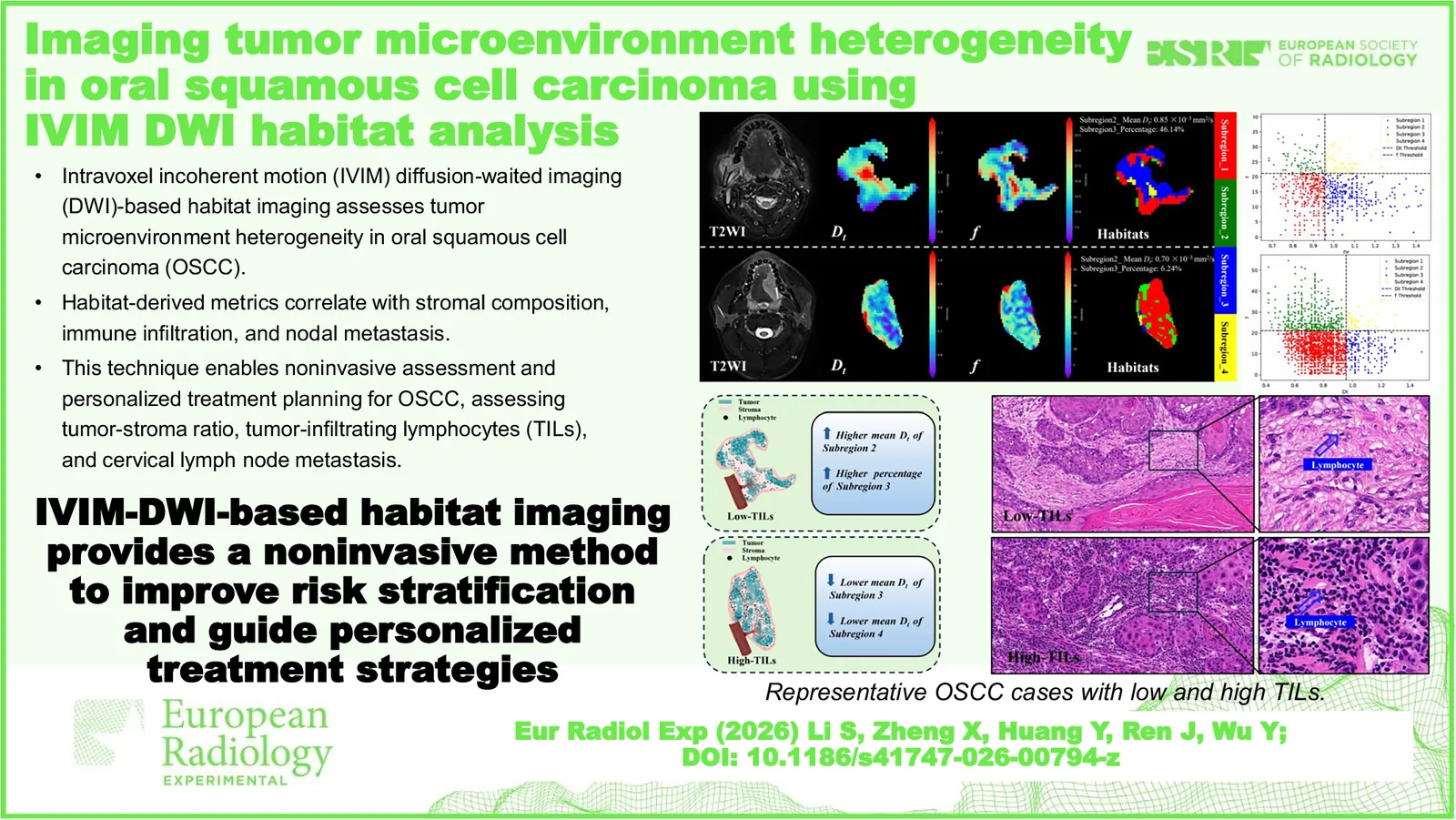

## Background

Oral squamous cell carcinoma (OSCC), accounting for nearly 90% of oral malignancies, remains a major global health burden, with an estimated 389,485 new cases and 188,230 related deaths in 2022 [1, 2]. OSCC commonly originates from the oral tongue, gingiva, oral floor, and cheek, and should be distinguished from oropharyngeal and tonsillar squamous cell carcinomas, which are often human papillomavirus-associated and biologically distinct [2]. Although the TNM staging system serves as the cornerstone for clinical staging and prognostic evaluation, patients with identical TNM stages often exhibit heterogeneous outcomes, reflecting its inability to fully capture tumor biological diversity [3].

Recent advances have highlighted the pivotal role of the tumor microenvironment (TME) in influencing tumor aggressiveness, metastatic potential, and therapeutic resistance [4]. Among histopathological biomarkers, the tumor-stroma ratio (TSR) and tumor-infiltrating lymphocytes (TILs) have emerged as key prognostic indicators, providing complementary insights into stromal remodeling and immune activation [5–7]. TSR, defined as the proportion of stromal tissue relative to tumor epithelium, has been recognized as a robust prognostic factor in OSCC [8]. A stroma-rich phenotype contributes to tumor progression by fostering extracellular matrix remodeling, immune suppression, and epithelial-mesenchymal transition, and is consistently linked to unfavorable survival outcomes [9]. Conversely, TILs represent antigen-specific immune cells capable of mounting antitumor responses. High TIL density in OSCC correlates with improved prognosis and enhanced responsiveness to immune checkpoint blockade [10–12]. Thus, TSR and TILs not only serve as prognostic biomarkers but also reflect the reciprocal interplay between stromal activation and immune surveillance, both central to personalized treatment decision-making in OSCC.

While histopathological evaluation remains the reference standard, biopsy-based assessment of TSR and TILs is invasive, prone to sampling bias, and not suitable for longitudinal monitoring. Consequently, there is a critical need for noninvasive imaging biomarkers that can comprehensively characterize the TME and dynamically track TSR and TIL variations during disease progression and treatment.

Magnetic resonance imaging (MRI) is routinely employed in OSCC owing to its superior soft tissue contrast and capability to delineate tumor invasion [13]. However, conventional anatomic imaging and diffusion-weighted imaging (DWI)-derived apparent diffusion coefficient (ADC) value offer only limited insights into biological heterogeneity, as ADC represents a composite of both diffusion and perfusion effects [14]. The intravoxel incoherent motion (IVIM)-DWI model overcomes this limitation by separating true diffusion (*D*_*t*_) from perfusion-related parameters (*D*_*p*_) and perfusion fraction (*f*), thereby enabling a more physiologically relevant assessment of tissue cellularity and micro-vascularity [15]. However, IVIM-DWI-based metrics are constrained by inherent model assumptions and insufficient spatial contextualization, which constrains their ability to fully characterize tumor microstructural heterogeneity [16].

Habitat imaging, an emerging multiparametric framework, addresses this limitation by partitioning tumors into biologically distinct subregions (“habitats”) based on diffusion and perfusion characteristics [17]. This strategy is grounded in the premise that spatially distinct combinations of quantitative imaging parameters reflect localized variations in tissue biology. By enabling both spatial visualization and volumetric quantification, habitat imaging allows a more comprehensive assessment of tumor composition and microenvironmental complexity [18]. Such region-specific analysis offers a promising avenue for TME phenotyping, potentially improving the prediction of key histopathological features (*e.g*., TSR and TILs), refining risk stratification, and supporting treatment monitoring. However, the application of IVIM-DWI–derived habitat imaging for histopathological characterization in OSCC remains largely unexplored.

Therefore, this study aims to apply an IVIM-DWI-based habitat imaging framework for quantitative evaluation of the TME in OSCC and to investigate its ability to predict TSR and TILs levels. By bridging spatially resolved imaging phenotypes with tumor-level pathological states, this study promotes the development of quantitative imaging biomarkers for TME characterization, enhances preoperative risk assessment, and facilitates more precise, personalized treatment strategies.

## Materials and methods

### Study population

This ongoing prospective study was approved by the ethical review board of our institute (No. SH9H-2023-T454-1). Informed consent was obtained from all individuals included in the study. We enrolled consecutive newly diagnosed patients with pathologically proven OSCC between June 2023 and May 2025. The inclusion criteria were: (1) patients with suspected oral cancers; (2) preoperative MRI performed prior to biopsy or surgical intervention; (3) no history of prior radiotherapy or chemotherapy. The exclusion criteria were: (1) absence of available pathological whole-slide specimens; (2) non-OSCC on pathological examination; (3) inadequate image quality or IVIM fitting failure; (4) tumors with a maximal diameter (MD) less than 10 mm.

### MRI acquisition

All patients underwent the MRI examination on a 3-T scanner (Magnetom Skyra, Siemens Healthineers, Erlangen, Germany) with a commercial 32-channel head and neck coil. The MRI protocols included: axial T1-weighted imaging (T1WI); axial and coronal T2-weighted imaging (T2WI) with fat suppression; axial, coronal, and sagittal contrast-enhanced T1WI (CE-T1WI). The IVIM-DWI acquisition was performed using single-shot spin-echo echo-planar imaging with three orthogonal motion-probing gradients and seven *b*-values (0, 50, 100, 200, 400, 800, and 1,500 s/mm²). Additional protocol parameters were for IVIM-DWI: echo time 77 ms, repetition time 2,100 ms, slice thickness/gap 4 mm/1.2 mm, field of view 245 × 245 mm^2^, matrix 384 × 384. IVIM-DWI was acquired using a single-shot spin-echo echo-planar imaging sequence. To minimize motion-related artifacts, patients were immobilized and instructed to avoid swallowing during acquisition.

### Image postprocessing and parametric map generation

Prior to offline analysis, all diffusion-weighted images with higher *b*-values (50–1,500 s/mm²) were rigidly registered to the *b* = 0 s/mm² image using the vendor-provided motion correction algorithm on the Siemens syngo.via workstation (Siemens Healthineers, Germany) to correct for intrascan motion and ensure spatial alignment across *b*-values. The registered images were subsequently imported into FireVoxel software (https://firevoxel.org/) for quantitative analysis. Tumor regions of interest (ROIs) were manually delineated on IVIM-DWI images (echo time = 77 ms, *b*-value = 800 s/mm²) by a radiologist with 3 years of head and neck imaging experience (S.L.). This *b*-value was selected because it provided optimal tumor-to-background contrast while minimizing perfusion-related signal contributions. ROIs encompassed all slices containing solid tumor components, and necrotic areas were excluded with reference to T2WI and CE-T1WI images.

IVIM model fitting was performed in a voxel-wise manner within tumor ROIs. Parametric maps of the true diffusion coefficient (*D*_*t*_) and perfusion fraction (*f*) were generated using the Parametric Map tool in the Dynamic Analysis module of FireVoxel according to the following equation:

$${S}_{(b)}={S}_{0}\times ((1-f)\times exp (-b\times {D}_{t})+f\times exp (-b\times {D}_{p}))$$

where *S*_*0*_ and *S*_*b*_ are the signal intensity of *b* = 0 s/mm^2^ and other b-values; *f*, *D*_*t*_ and *D*_*p*_ are the abbreviations of perfusion fraction, true diffusion coefficient, and pseudo diffusion coefficient. *f* represents the intravoxel volume fraction of perfusion. *D*_*p*_ and *D*_*t*_ represent perfusion-related diffusion and pure diffusion.

Cases with severe geometric distortion, signal loss, or unstable IVIM model fitting were excluded from further analysis. In addition to IVIM-DWI-based parameters, conventional imaging metrics such as MD and depth of invasion (DOI) was measured. MD was measured as the largest tumor dimension on axial, coronal, and sagittal CE-T1WI images. DOI was assessed on axial CE-T1WI by drawing a perpendicular line from the basement membrane of the adjacent intact mucosa to the deepest point of tumor infiltration [19]. Furthermore, the whole-tumor mean ADC values were extracted from conventional ADC maps (*b* = 0 and 800 s/mm²) to serve as a reference parameter, given their widespread clinical use in head and neck cancer evaluation.

### Habitat clustering and parameter extraction

The habitat subregions were generated, and features were extracted using Python (https://www.python.org/). Whole-tumor ROIs from all patients were pooled, and voxel-wise *D*_*t*_ and *f* values were clustered at the population level using the Otsu thresholding method to define four imaging habitats. The Otsu method iteratively determines optimal threshold values by minimizing within-class variance while maximizing between-class variance [20]. The quantitative image features included the percentage, volume, mean *D*_*t*_, and mean *f* values for each habitat, as well as mean *D*_*t*_ and *f* values of the whole tumor.

To evaluate the interobserver reproducibility of habitat parameters, 20 randomly selected OSCC cases were independently delineated on the same IVIM-DWI reference images (echo time = 77 ms, *b* = 800 s/mm²) using FireVoxel software by a second radiologist with 10 years of experience in head and neck imaging (J.R.). Interobserver reproducibility was assessed using the two-way random effects intraclass correlation coefficient (ICC) for absolute agreement. ICCs were interpreted as follows: poor, < 0.4; fair, 0.4–0.59; good, 0.6–0.75; excellent, > 0.75 [21].

### Histopathology evaluation

After surgical resection, tumor specimens were fixed in formalin and stained with hematoxylin and eosin and subsequently digitized using the Pannoramic microscope scanner (3DHISTECH Ltd., Budapest, Hungary). For each patient, one to three representative whole-slide sections from the largest cross-sectional tumor area were selected for pathological evaluation. These sections were required to include both the main tumor body and the invasive front, in accordance with routine diagnostic practice in OSCC. All pathological evaluations were initially reported by one pathologist (X.Z., with 6 years of experience in oral pathology) in a blinded manner and subsequently reviewed and confirmed by a senior pathologist (Y.H., with 10 years of experience in oral pathology). TSR was evaluated at the invasive front by estimating the proportion of stromal tissue relative to tumor epithelium. Tumors were categorized as low TSR (< 50% stroma) or high TSR (≥ 50% stroma), consistent with previously established criteria in OSCC [3]. TILs were evaluated using the scoring method introduced by the International Immuno-Oncology Biomarkers Working Group [22]. TILs were quantified as the percentage of stromal area occupied by mononuclear inflammatory cells (excluding necrotic regions and non-tumor-associated stroma), evaluated by averaging percentages in various stromal areas on whole-slide sections [23]. Patients were dichotomized into high- and low-TILs groups using the cohort median stromal TILs percentage (40%). CLNM status was determined based on postoperative pathologic examination. TSR, TILs, and CLNM were defined as tumor-level pathological phenotypes reflecting global stromal abundance, immune infiltration, and metastatic propensity. Accordingly, these variables were correlated with whole-tumor imaging-derived habitat metrics rather than spatially matched subregions. Direct slice-by-slice radiologic-pathologic correspondence was not attempted due to inherent differences in spatial resolution and tissue deformation following surgical resection.

### Statistical analysis

Statistical analyses were conducted using R software (https://www.r-project.org/). Data normality was evaluated with the Shapiro–Wilk test. Continuous variables with normal distribution were presented as mean ± standard deviation (mean ± standard deviation), those with non-normal distribution as median (Q1, Q3), and categorical variables as frequencies and percentages. Differences between low and high TSR and TILs groups, as well as between CLNM-negative and CLNM-positive groups, were assessed using the chi-square, independent *t*-test, or Mann–Whitney *U* test. Univariate and stepwise multivariate regression analyses were used to identify metrics associated with TSR and TILs, and independent predictors were incorporated into nomogram models. Calibration curves were generated to evaluate agreement between predicted and observed outcomes. Multicollinearity among the independent variables was assessed using the variance inflation factor in the linear regression model. Predictive performance of individual predictors and habitat-based models was assessed by receiver operating characteristic (ROC) analysis. The area under the curve (AUC), accuracy, sensitivity, and specificity with 95% confidence intervals (CIs) were calculated to quantify the predictive efficacy. Five-fold cross-validation was performed to mitigate model overfitting. Correlations between MRI-derived metrics and pathological parameters were analyzed using Spearman (ρ), point-biserial (ρ), and Phi (φ) coefficients, as appropriate. A *p*-value < 0.05 was considered statistically significant.

## Results

### Study population and baseline characteristics

**Fig. 1:**
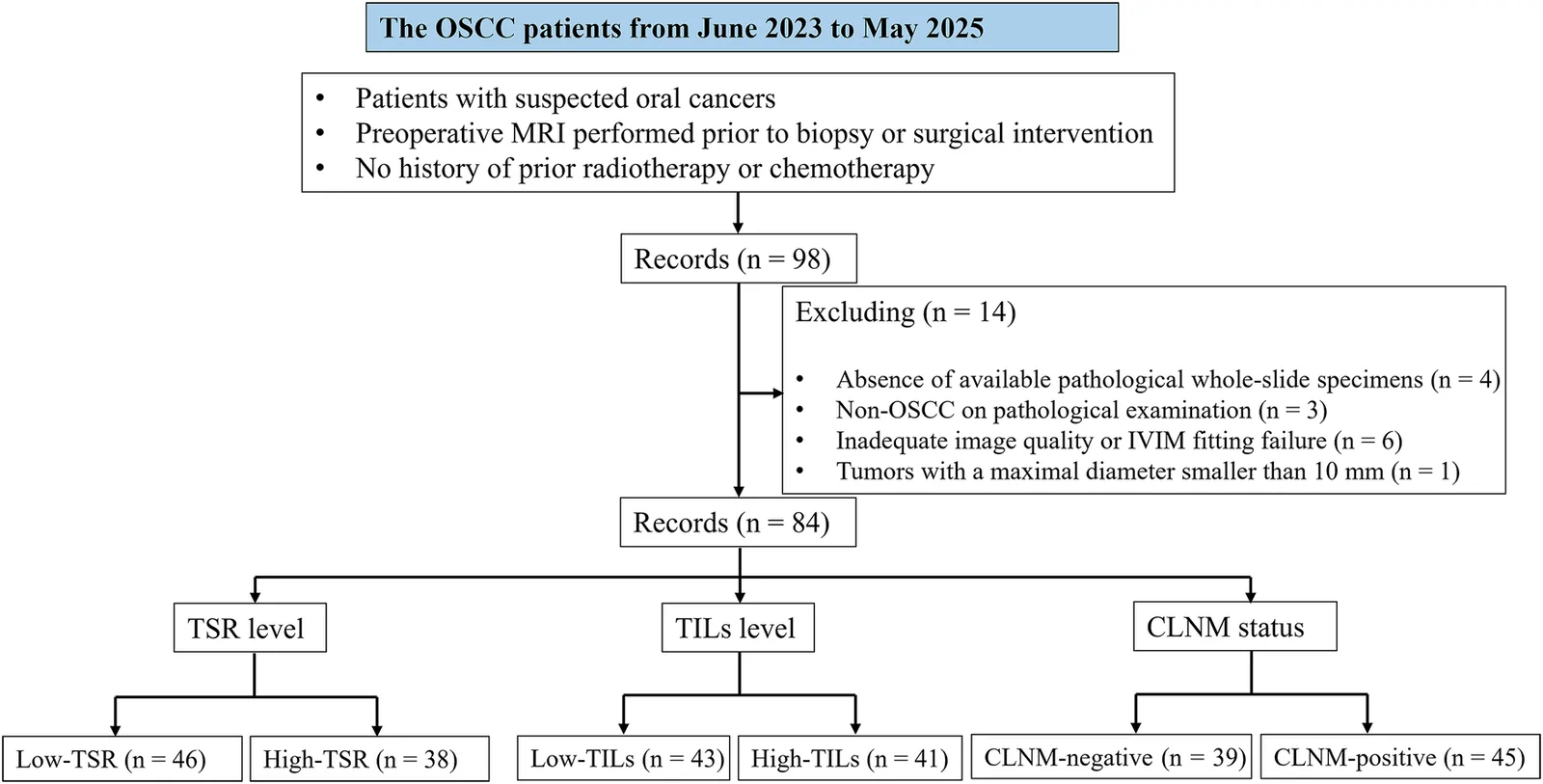
Flowchart of the patient inclusion process. CLNM, Cervical lymph node metastasis; IVIM, Intravoxel incoherent motion; OSCC, Oral squamous cell carcinoma; TILs, Tumor-infiltrating lymphocytes; TSR, Tumor-stroma ratio

A total of 84 patients with OSCC were enrolled in this study (Fig. 1). Among them, 45% were classified as high TSR, 49% as high TILs, and 54% were pathologically confirmed as CLNM-positive. Detailed demographic and pathological characteristics stratified by TSR, TILs, and CLNM status are presented in Tables 1 and S1, respectively. Baseline clinical characteristics were largely comparable between the low- and high-TSR and TILs groups. The only significant difference was a greater DOI in the high-TSR group. No significant differences were observed in age, sex, tumor subsite, clinical T stage, CLNM status, or MD. In contrast, patients with CLNM-positive exhibited significantly larger tumors and deeper invasion compared with those without nodal metastasis. Advanced clinical T stages were also more frequently observed in the CLNM-positive group.

**Table 1:** Comparison of clinical and pathological characteristics between different TSR and TIL groups

	TSR			TILs		
	Low-TSR (*n* = 46)	High-TSR (*n* = 38)	*p*^1^-value	Low-TILs (*n* = 43)	High-TILs (*n* = 41)	*p*^2^*-*value
Age (years)	55.5 ± 17.5	57.3 ± 13.6	0.602	56.2 ± 14.5	56.5 ± 17.3	0.915
Sex (*n*, %)			0.170			0.953
Male	26 (56.5)	27 (71.1)		27 (62.8)	26 (63.4)	
Female	20 (43.5)	11 (28.9)		16 (37.2)	15 (36.6)	
Subsite (*n*, %)			0.264			0.853
Tongue	43 (93.5)	32 (84.2)		37 (86.0)	38 (92.7)	
Oral floor	2 (4.3)	1 (2.6)		2 (4.7)	1 (2.4)	
Cheek	0 (0.0)	2 (5.3)		1 (2.3)	1 (2.4)	
Gingiva	1 (2.2)	3 (7.9)		3 (7.0)	1 (2.4)	
cT stage (*n*, %)			0.170			0.194
T1-2	20 (43.5)	11 (28.9)		13 (30.2)	18 (43.9)	
T3-4	26 (56.5)	27 (71.1)		30 (69.8)	23 (56.1)	
CLNM			0.109			0.083
N0	25 (54.3)	14 (36.8)		16 (37.2)	23 (56.1)	
N1-3	21 (45.7)	24 (63.2)		27 (62.8)	18 (43.9)	
MD (cm)	2.7 ± 1.0	3.2 ± 1.4	0.055	3.0 ± 1.4	2.8 ± 1.1	0.337
DOI (cm)	1.3 ± 0.6	1.7 ± 1.0	**0.033**	1.5 ± 0.9	1.4 ± 0.8	0.410

Data are expressed as mean ± standard deviation or number (percentage). *p*^1^: Low-TSR *versus* High-TSR; *p*^2^: Low TILs *versus* High TILs. Bold denotes statistically significant results (*p* < 0.05)

*CLNM* Cervical lymph node metastasis, *cT stage* Clinical T stage, *DOI* Depth of invasion, *MD* Maximal diameter, *OSCC* Oral squamous cell carcinoma, *TSR* Tumor-stroma ratio, *TILs* Tumor-infiltrating lymphocytes

### Illustration of IVIM-DWI-based habitat cluster and metrics extraction

**Fig. 2:**
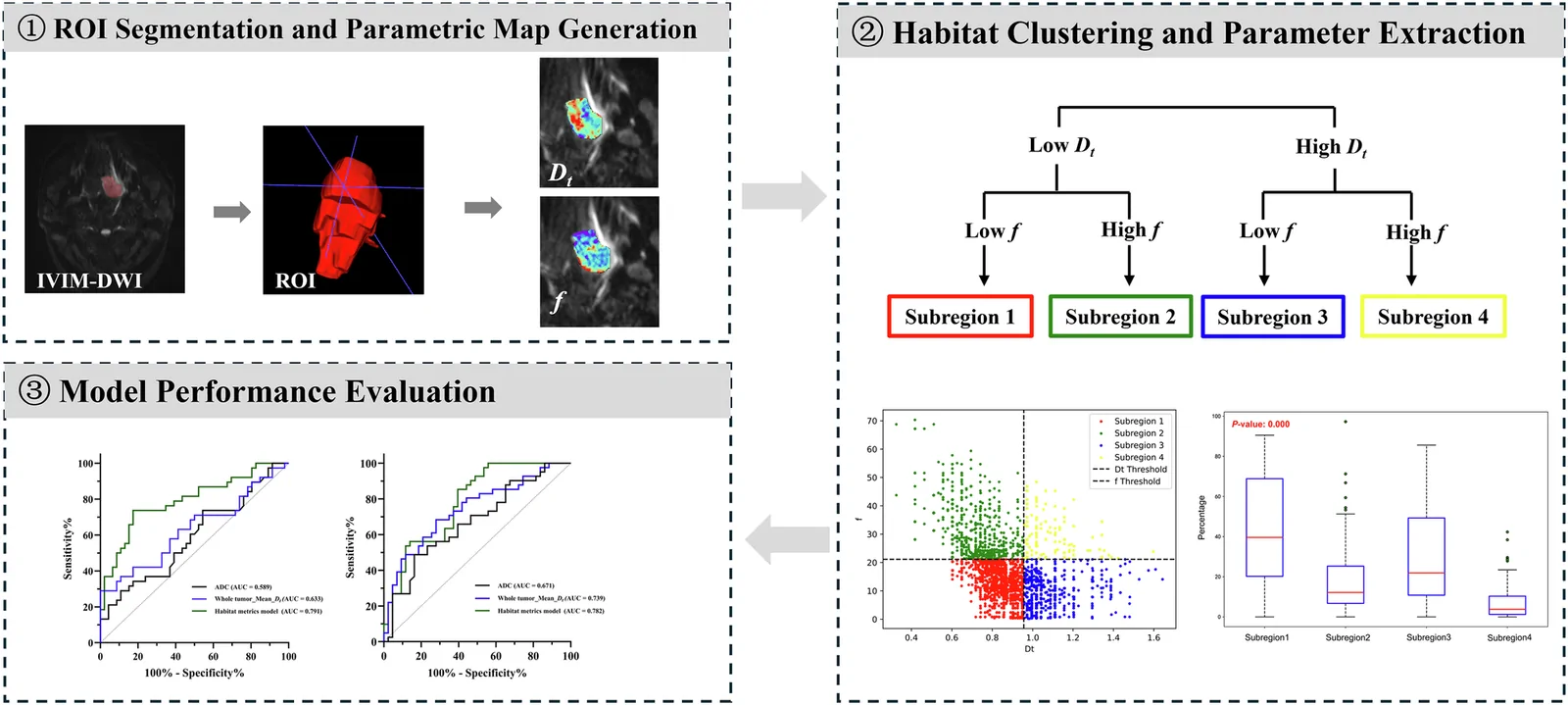
Schematic workflow of the study. IVIM-DWI, Intravoxel incoherent motion-diffusion-weighted imaging; ROI, Region of interest

Based on the voxel-wise distribution of *D*_*t*_ and *f* values, tumors were partitioned into four subregions reflecting distinct diffusion–perfusion patterns (Fig. 2). Subregion 1 was characterized by low *D*_*t*_ and low *f* values and may represent relatively hypercellular and hypoperfused compartments, potentially compatible with densely packed tumor regions or areas with limited microvascular supply. Subregion 2 demonstrated low *D*_*t*_ and high *f* values, suggesting hypercellular regions with relatively increased perfusion, which may be consistent with immune-enriched or metabolically active tumor compartments. Subregion 3 exhibited high *D*_*t*_ and low *f* values, a pattern that could reflect hypocellular microenvironments with expanded extracellular space, possibly corresponding to stromal-rich regions. Subregion 4 showed high *D*_*t*_ and high *f* values, suggestive of relatively loose cellular architecture accompanied by elevated perfusion, which may be compatible with tumor margins or invasive fronts. Importantly, these biologically informed descriptors were intended to facilitate physiological interpretation of habitat-derived metrics rather than imply direct histopathological equivalence.

### Association between habitat imaging metrics and pathological TSR, TILs, and CLNM

**Fig. 3:**
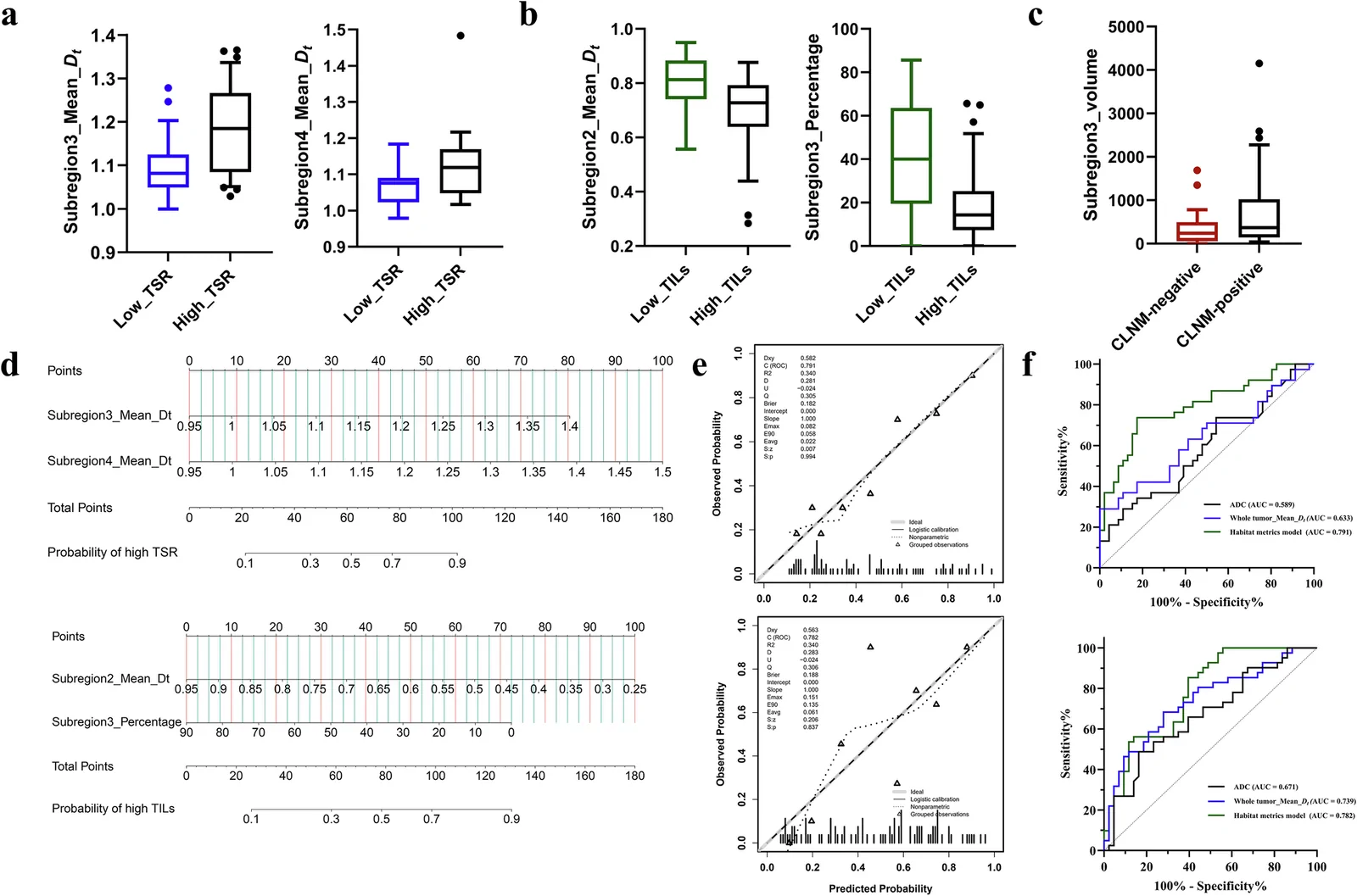
Box-and-whisker plots illustrating the independent predictive habitat parameters distinguishing (**a**) low and high TSR groups; (**b**) low and high TILs groups; and (**c**) CLNM-negative and CLNM-positive groups. **d**–**f** Nomogram for predicting TSR and TILs levels in OSCC and its calibration and ROC curves. CLNM, Cervical lymph node metastasis; OSCC, Oral squamous cell carcinoma; TILs, Tumor-infiltrating lymphocytes; TSR, Tumor-stroma ratio; ROI, Region of interest

As shown in the reproducibility analysis (Table S2), all habitat metrics exhibited excellent interobserver agreement (ICCs > 0.75). Exploratory comparisons across groups with different TSR, TILs, and CLNM status were conducted to investigate differences in the spatial distribution and microstructural patterns of IVIM-DWI-derived habitats (Table 2). Tumors with higher TSR level exhibited significantly increased mean *D*_*t*_ values in subregions 3 and 4 (1.18 *versus* 1.08 × 10⁻³ mm²/s, *p* < 0.001; 1.12 *versus* 1.08 × 10⁻³ mm²/s, *p* = 0.001, respectively) (Fig. 3 and Table 2). In Table 3, univariate analysis indicated strong associations between TSR and the mean *D*_*t*_ value of the whole tumor as well as subregions 3 and 4. Multivariate analysis further confirmed that the mean *D*_*t*_ value of subregion 3 (odds ratio (OR): 2.493, 95% CI: 1.353–4.592, *p* = 0.003) and subregion 4 (OR: 2.054, 95% CI: 1.022–4.131, *p* = 0.043) remained independent predictors of high TSR, suggesting that tumors with higher stromal content are characterized by increased extracellular space and reduced cellular packing.

**Table 2:** Comparison of habitat-derived parameters between low and high TSR and TILs groups

	TSR			TILs			CLNM		
	Low-TSR (*n* = 46)	High-TSR (*n* = 38)	*p*^1^-value	Low-TILs (*n* = 43)	High-TILs (*n* = 41)	*p*^2^-value	CLNM-negative (*n* = 39)	CLNM-positive (*n* = 45)	*p*^3^-value
Whole Tumor_ADC (10^-3^ mm^2^/s)	1.02 (0.96, 1.09)	1.06 (0.96, 1.13)	0.164	1.07 (1.00, 1.13)	0.99 (0.92, 1.08)	**0.007**	1.07 (0.97, 1.11)	1.02 (0.95, 1.08)	0.335
Whole Tumor_Mean_*D*_*t*_ (10^-3^ mm^2^/s)	0.86 (0.77, 0.99)	0.91 (0.80, 1.14)	**0.037**	0.98 (0.86, 1.09)	0.84 (0.75, 0.91)	**< 0.001**	0.90 (0.77, 1.08)	0.87 (0.80, 1.00)	0.412
Whole Tumor_Mean_*f* (%)	14.25 (11.63, 18.54)	14.44 (11.80, 16.72)	0.986	14.12 (11.55, 16.40)	15.07 (11.81, 19.82)	0.205	13.91 (11.40, 21.03)	14.68 (11.95, 17.64)	0.819
Subregion1_Percentage (%)	41.90 (26.74, 68.86)	38.48 (7.21, 70.42)	0.166	36.27 (12.07, 59.60)	51.94 (30.31, 74.97)	0.054	28.19 (11.74, 65.99)	45.31 (30.55, 71.89)	0.113
Subregion1_Volume (mm^3^)	547 (134, 1,288)	521 (51, 1,684)	0.893	333 (108, 1,351)	677 (151, 1,295)	0.505	243 (55, 965)	1,046 (164, 2,215)	**0.002**
Subregion1_Mean_ *D*_*t*_ (10^-3^ mm^2^/s)	0.83 (0.76, 0.87)	0.83 (0.78, 0.87)	0.978	0.85 (0.82, 0.89)	0.80 (0.74, 0.84)	**< 0.001**	0.83 (0.79, 0.87)	0.82 (0.76, 0.87)	0.551
Subregion1_Mean_*f* (%)	11.57 (9.77, 13.01)	11.57 (10.31, 12.19)	0.975	11.66 (9.54, 13.03)	11.38 (9.96, 12.81)	0.741	11.57 (9.83, 12.99)	11.66 (9.68, 12.92)	0.971
Subregion2_Percentage (%)	15.11 (6.52, 33.56)	11.76 (6.49, 17.66)	0.239	9.74 (6.13, 15.27)	16.74 (8.76, 35.83)	**0.003**	11.29 (6.78, 32.61)	12.55 (6.46, 26.03)	0.760
Subregion2_Volume (mm^3^)	164 (63, 301)	164 (75, 444)	0.653	150 (65, 303)	176 (80, 399)	0.293	102 (38, 214)	278 (85, 553)	**0.003**
Subregion2_Mean_ *D*_*t*_ (10^-3^ mm^2^/s)	0.77 (0.69, 0.85)	0.76 (0.69, 0.84)	0.854	0.81 (0.74, 0.88)	0.73 (0.64, 0.79)	**< 0.001**	0.77 (0.65, 0.84)	0.75 (0.70, 0.84)	0.723
Subregion2_Mean_f (%)	27.41 (25.67, 31.49)	29.17 (26.68, 34.45)	0.088	27.84 (25.83, 32.79)	27.84 (26.41, 33.67)	0.480	27.84 (25.59, 34.40)	27.90 (26.48, 32.24)	0.968
Subregion3_Percentage (%)	19.12 (9.79, 38.77)	32.58 (10.78, 63.70)	0.111	40.00 (19.46, 63.64)	14.29 (7.33, 25.32)	**< 0.001**	23.08 (11.45, 56.86)	19.46 (9.61, 44.11)	0.597
Subregion3_Volume (mm^3^)	217 (61, 561)	354 (202, 791)	0.051	376 (211, 1,092)	167 (55, 493)	**0.001**	241 (55, 494)	370 (145, 1,022)	**0.019**
Subregion3_Mean_ *D*_*t*_ (10^-3^ mm^2^/s)	1.08 (1.05, 1.12)	1.18 (1.08, 1.27)	**< 0.001**	1.13 (1.07, 1.20)	1.09 (1.05, 1.19)	0.122	1.11 (1.07, 1.19)	1.10 (1.06, 1.19)	0.690
Subregion3_Mean_ *f* (%)	10.01 (8.43, 11.66)	10.02 (7.82, 11.12)	0.523	10.04 (8.53, 11.62)	9.98 (7.12, 11.60)	0.390	9.87 (7.71, 11.57)	10.16 (8.49, 11.63)	0.344
Subregion4_Percentage (%)	3.15 (0.79, 7.56)	6.15 (1.62, 15.13)	**0.013**	5.93 (1.41, 15.11)	2.82 (0.79, 6.55)	**0.011**	5.04 (1.16, 11.59)	3.57 (1.21, 9.96)	0.812
Subregion4_Volume (mm^3^)	38 (5, 100)	78 (37, 149)	**0.007**	76 (30, 167)	40 (8, 96)	**0.012**	46.57 (10.58, 86.79)	76.20 (29.63, 168.28)	**0.015**
Subregion4_Mean_ *D*_*t*_ (10^-3^ mm^2^/s)	1.08 (1.02, 1.09)	1.12 (1.05, 1.17)	**0.001**	1.09 (1.05, 1.14)	1.08 (1.04, 1.10)	0.174	1.08 (1.03, 1.16)	1.09 (1.05, 1.14)	**0.043**
Subregion4_Mean_ *f* (%)	26.98 (25.72, 28.81)	27.54 (25.89, 32.82)	0.100	26.98 (25.83, 29.97)	26.98 (25.89, 32.54)	0.714	26.98 (25.83, 31.23)	26.98 (25.87, 31.59)	0.865

Data are expressed as median (25th, 75th quartiles). Mann–Whitney *U* test to compare the difference of habitat-derived parameters between each two groups. *p*^1^: Low-TSR *versus* High-TSR; *p*^2^: Low-TILs *versus* High-TILs; *p*^3^: CLNM-negative *versus* CLNM-positive. Bold denotes statistically significant results (*p* < 0.05)

*ADC* Apparent diffusion coefficient, *CLNM* Cervical lymph node metastasis, *TSR* Tumor-stroma ratio, *TILs* Tumor-infiltrating lymphocytes

**Table 3:** Univariate and multivariate logistic regression analyses of habitat-derived metrics for TSR, TILs, and CLNM

	Univariate analysis		Multivariate analysis	
	Odds ratio (95% CI)	*p-*value	Odds ratio (95% CI)	*p-*value
TSR				
Whole Tumor_Mean_*D*_*t*_	1.720 (1.075, 2.751)	**0.024**		
Subregion3_Mean_*D*_*t*_	3.162 (1.755, 5.699)	**< 0.001**	2.493 (1.353, 4.592)	**0.003**
Subregion4_Mean_*D*_*t*_	2.919 (1.539, 5.537)	**0.001**	2.054 (1.022, 4.131)	**0.043**
TILs				
Whole Tumor_Mean_*D*_*t*_	0.387 (0.224, 0.668)	**< 0.001**		
Subregion1_Mean_*D*_*t*_	0.370 (0.202, 0.677)	**0.001**		
Subregion1_Percentage	1.587 (1.012, 2.488)	**0.044**		
Subregion2_Percentage	2.301 (1.272, 4.161)	**0.006**		
Subregion2_Mean_*D*_*t*_	0.318 (0.165, 0.614)	**< 0.001**	0.461 (0.233, 0.912)	**0.026**
Subregion3_Percentage	0.336 (0.191, 0.593)	**< 0.001**	0.446 (0.245, 0.814)	**0.009**
Subregion3_volume	0.387 (0.191, 0.783)	**0.008**		
Subregion4_volume	0.436 (0.191, 0.997)	**0.049**		
CLNM				
Subregion1_volume	3.162 (1.130, 8.851)	**0.028**		
Subregion3_volume	2.434 (1.212, 4.888)	**0.012**	2.434 (1.212, 4.888)	**0.012**
Subregion4_volume	4.157 (1.263, 13,678)	**0.019**		

Bold denotes statistically significant results (*p* < 0.05)

*CI* Confidence interval, *CLNM* Cervical lymph node metastasis, *TSR* Tumor-stroma ratio, *TILs* Tumor-infiltrating lymphocytes

Conversely, tumors with high TIL levels displayed decreased mean *D*_*t*_ value in subregion 2 and a markedly lower percentage of subregion 3 (0.73 *versus* 0.81 × 10⁻³ mm²/s, *p* < 0.001; 14.29% *versus* 40.00%, *p* < 0.001, respectively) (Fig. 3 and Table 2). In Table 3, univariate analysis revealed that high-TIL tumors were characterized by decreased mean *D*_*t*_ value in both the whole tumor and cellular-dominant subregions 1–2, accompanied by an increased spatial prevalence of these habitats. In contrast, subregions 3–4 exhibited both reduced proportional representation and smaller volumes. On multivariate analysis, the mean *D*_*t*_ of subregion 2 (OR: 0.461, 95% CI: 0.233–0.912, *p* = 0.026) and the percentage of subregion 3 (OR: 0.446, 95% CI: 0.245–0.814, *p* = 0.009) emerged as independent predictors of TILs level. Collectively, this habitat pattern suggests a microenvironment characterized by higher cellular density and constrained diffusion alongside relative stromal contraction, a configuration that is biologically compatible with heightened lymphocytic infiltration.

Regarding CLNM status, patients with positive CLNM exhibited overall enlargement across all four subregions (Table 2), indicating a global expansion of heterogeneous tumor habitats. Notably, the volume of subregion 3 (370 *versus* 241 mm³, *p* = 0.019) showed the strongest association with positive CLNM. In Table 3, univariate analysis revealed that larger volumes of subregions 1, 3, and 4 correlated significantly with CLNM positivity, whereas multivariate regression analysis identified only the volume of stroma-dominant habitat (subregion 3) (OR: 2.434, 95% CI: 1.212–4.888, *p* = 0.012) as an independent predictor for CLNM, highlighting its potential relevance to metastatic propensity. To further explore whether habitat metrics captured biologically meaningful characteristics rather than merely reflecting tumor burden, we compared the predictive performances of clinical, habitat-only, and combined models. As shown in Table S3, the habitat-only model demonstrated slightly superior discriminative ability compared with the clinical baseline model (AUC: 0.732 *versus* 0.702; accuracy: 0.702 *versus* 0.679). Importantly, integrating habitat and clinical variables yielded a modest improvement in discrimination (AUC = 0.748), although overall accuracy (0.690) remained comparable to that of the individual models.

### Predictive performance of habitat-based models

The variance inflation factor values were 1.236 for the mean *D*_*t*_ of subregions 3 and 4, and 1.247 for the mean *D*_*t*_ of subregion 2 and the percentage of subregion 3, suggesting no multicollinearity concerns in constructing the habitat metrics model. To assess the incremental predictive value of IVIM-DWI-derived habitat metrics, conventional whole-tumor ADC and mean *D*_*t*_ values were evaluated for the prediction of TSR and TILs levels, respectively. Whole-tumor ADC value showed limited discriminatory performance for TSR and TILs (AUC = 0.589, accuracy = 0.583; AUC = 0.671, accuracy = 0.655), whereas whole-tumor mean *D*_*t*_ achieved moderate improvement (AUC = 0.633, accuracy = 0.679; AUC = 0.739, accuracy = 0.702). In contrast, the integrated habitat metrics model consistently outperformed conventional whole-tumor metrics, achieving superior discrimination for both TSR (AUC = 0.791, accuracy = 0.786) and TILs (AUC = 0.782, accuracy = 0.726), underscoring the added value of habitat-based analysis for capturing intratumoral heterogeneity (Table 4). Five-fold cross-validation further confirmed the stability and generalizability of the habitat models, yielding a mean AUC of 0.826 (95% CI: 0.644–1.000) for TSR prediction and 0.777 (95% CI: 0.634–0.920) for TILs prediction (Table S4 and Fig. S1).

**Table 4:** Predictive performance of univariate and habitat-based models for TSR and TILs levels

	AUC (95% CI)	Sensitivity (95% CI)	Specificity (95% CI)	Accuracy (95% CI)	Cut-off
Prediction of TSR level					
Whole tumor_ADC (10^-3^ mm^2^/s)	0.589 (0.465, 0.712)	0.737 (0.569, 0.866)	0.457 (0.309, 0.610)	0.583 (0.471, 0.690)	0.996
Whole tumor_Mean_*D*_*t*_ (10^-3^ mm^2^/s)	0.633 (0.510, 0.755)	0.289 (0.154, 0.459)	1.000 (0.923, 1.000)	0.679 (0.568, 0.776)	1.12
Habitat metrics model	0.791 (0.692, 0.890)	0.737 (0.569, 0.866)	0.826 (0.686, .922)	0.786 (0.683, 0.868)	/
Prediction of TILs level					
Whole tumor_ADC (10^-3^ mm^2^/s)	0.671 (0.555, 0.786)	0.837 (0.693, 0.932)	0.488 (0.329, 0.649)	0.655 (0.555, 0.766)	0.982
Whole tumor_Mean_*D*_*t*_ (10^-3^ mm^2^/s)	0.739 (0.632, 0.846)	0.721 (0.563, 0.847)	0.683 (0.519, 0.819)	0.702 (0.593, 0.797)	0.883
Habitat metrics model	0.782 (0.684, 0.879)	0.854 (0.708, 0.944)	0.605 (0.444, 0.750)	0.726 (0.618, 0.812)	/

*ADC* Apparent diffusion coefficient, *AUC* Area under the curve, *CI* Confidence interval, *TSR* Tumor-stroma ratio, *TILs* Tumor-infiltrating lymphocytes

**Fig. 4:**
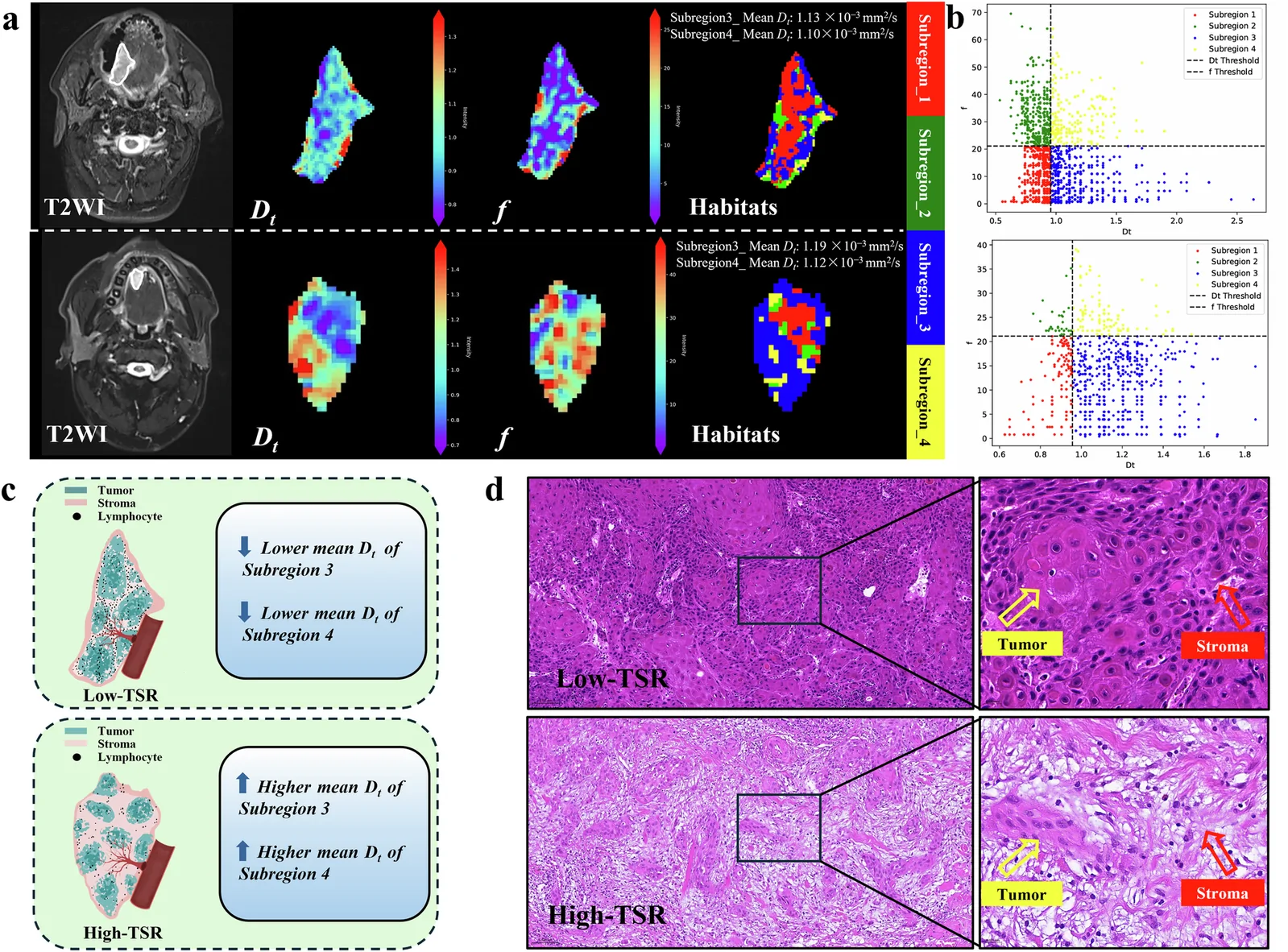
Representative cases with low and high TSR. **a** Axial T2WI images, *D*_*t*_ maps, *f* maps, and corresponding overlaid habitat maps of the oral tongue cancer (white circle). Each color denotes a distinct tumor habitat: subregion 1 (red), subregion 2 (green), subregion 3 (blue), and subregion 4 (yellow). **b** Scatter plots showing the distribution of *D*_*t*_ and *f* values for all pixels within each habitat. **c** Schematic illustrations depicting the histologic and habitat-based characteristics of low and high TSR tumors. **d** H&E sections of tumors with low and high TSR (yellow arrows, tumor cells; red arrows, stroma). H&E, Hematoxylin and eosin; T2WI, T2-weighted imaging; TILs, Tumor-infiltrating lymphocytes; TSR, Tumor-stroma ratio

**Fig. 5:**
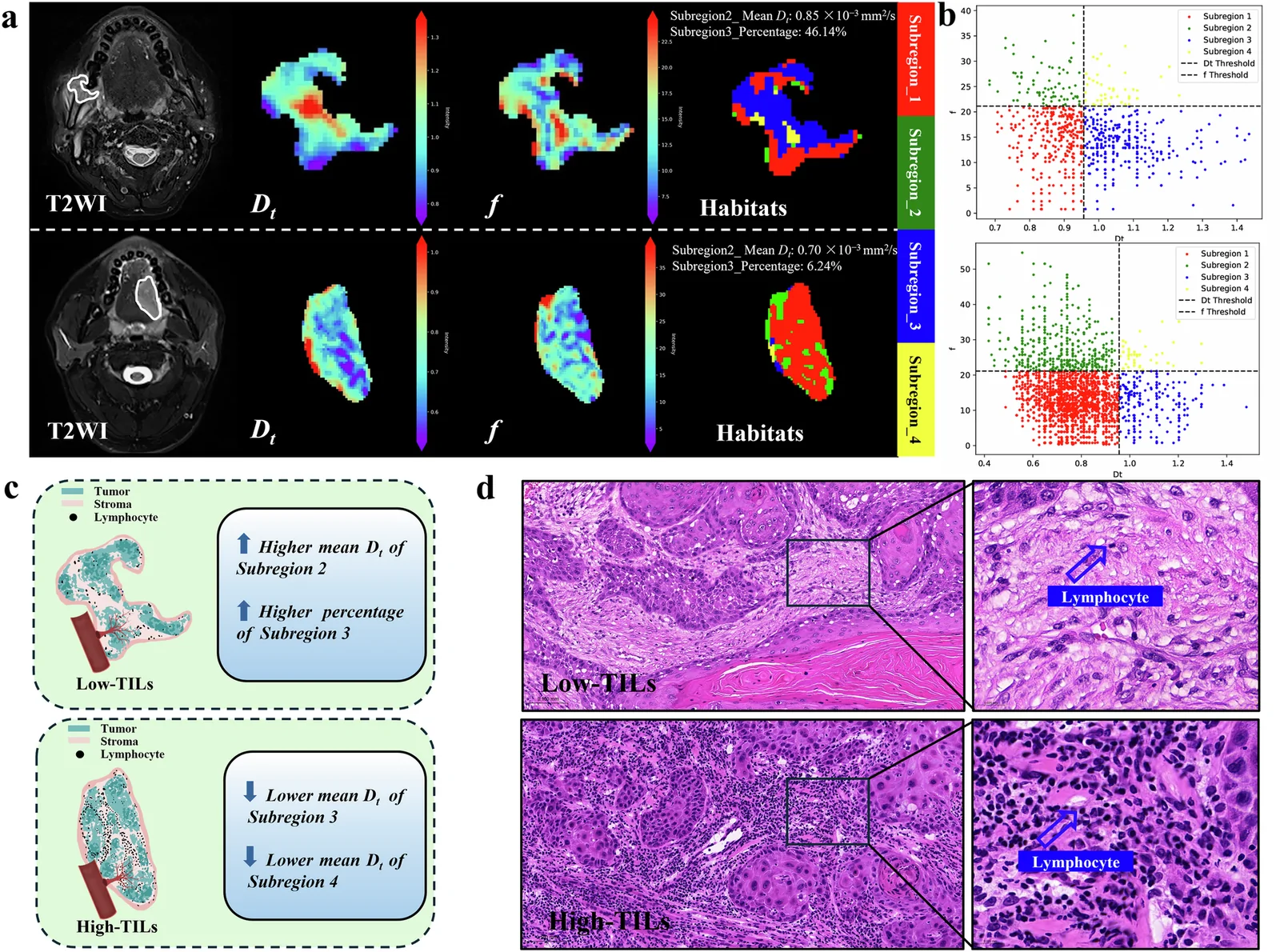
Representative cases with low and high TILs. **a** Axial T2WI images, *D*_*t*_ maps, *f* maps, and corresponding overlaid habitat maps of the gingiva cancer (white circle). Each color denotes a distinct tumor habitat: subregion 1 (red), subregion 2 (green), subregion 3 (blue), and subregion 4 (yellow). **b** Scatter plots showing the distribution of *D*_*t*_ and *f* values for all pixels within each habitat. **c** Schematic illustrations depicting the histologic and habitat-based characteristics of low and high-TIL tumors. **d** H&E sections of tumors with low and high TILs (blue arrows, lymphocytes). H&E, Hematoxylin and eosin; T2WI, T2-weighted imaging; TILs, Tumor-infiltrating lymphocytes; TSR, Tumor-stroma ratio

The corresponding nomogram and ROC analyses of the predictive models are displayed in Fig. 3. Nomogram models based on independent imaging predictors from multivariate analysis allow individualized preoperative estimation of TSR and TILs in OSCC, providing a quantitative tool to assess tumor-stroma composition and immune infiltration for personalized treatment planning and prognostic stratification. Representative OSCC cases with low and high TSR and TILs are shown in Figs. 4 and 5.

### Correlation between habitat metrics and clinical-pathological features

**Fig. 6:**
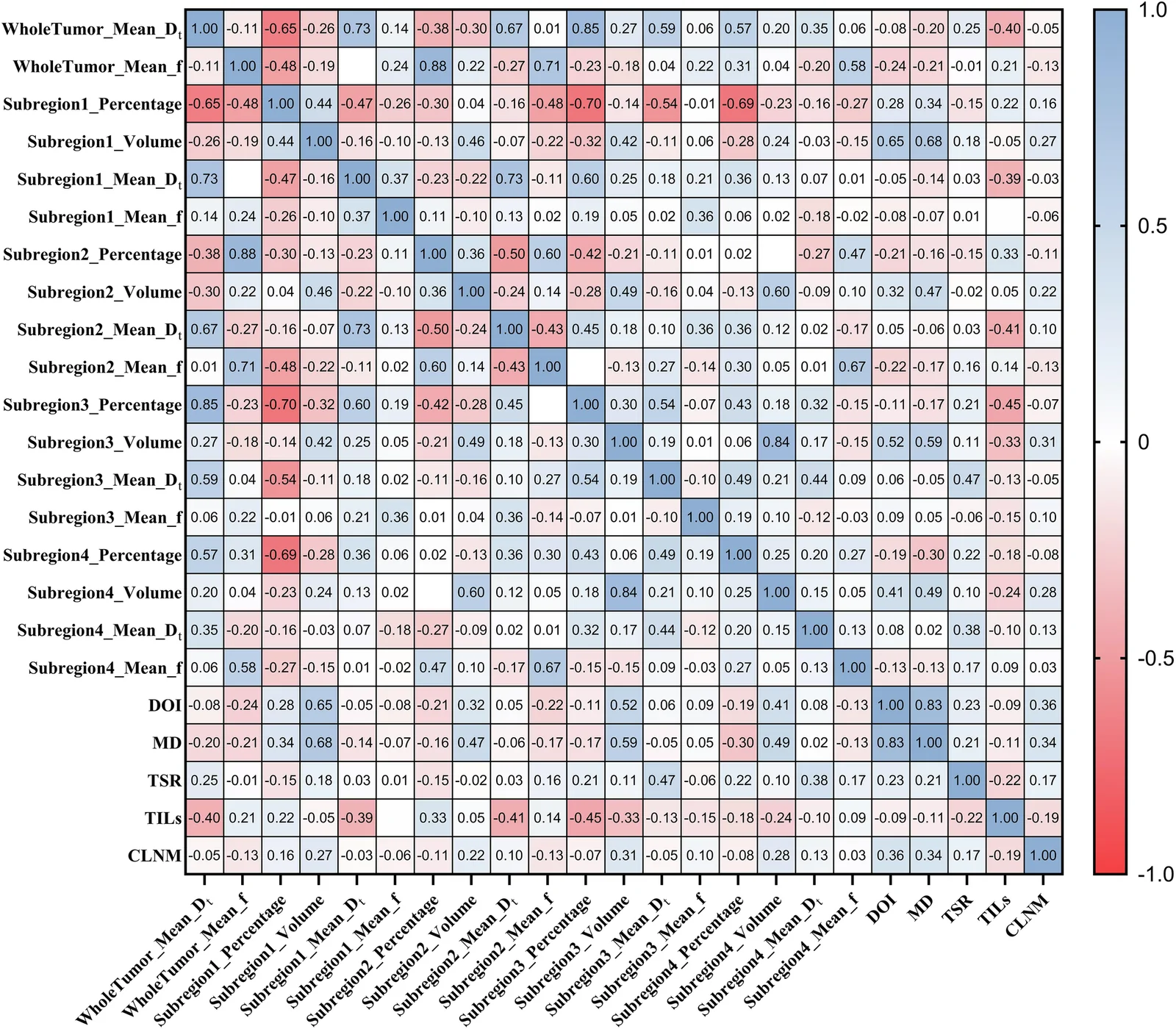
Correlation analysis between imaging and pathologic metrics. CLNM, Cervical lymph node metastasis; DOI, Depth of invasion; MD, Maximal diameter; OSCC, Oral squamous cell carcinoma; TILs, Tumor-infiltrating lymphocytes; TSR, Tumor-stroma ratio

Correlation analyses revealed that several IVIM-DWI-derived habitat metrics were significantly associated with key pathological characteristics and clinical outcomes (Fig. 6). Consistent with the findings from univariate and multivariate analysis, TSR showed positive correlations with the mean *D*_*t*_ of subregion 3 and subregion 4 (ρ = 0.47, ρ = 0.38, respectively; both *p* < 0.001); In contrast, TILs exhibited negative correlations with the mean *D*_*t*_ of the whole tumor and subregions 1–2 (*ρ* = -0.40, -0.39, and -0.41; all *p* < 0.001), as well as with the percentage of subregion 3 (ρ = -0.45, *p* < 0.001) and the volumes of subregions 3 and 4 (ρ = -0.33, -0.24; *p* = 0.002, 0.031, respectively). In addition, TSR was negatively correlated with TILs (φ = -0.22, *p* = 0.047), whereas CLNM showed a weak positive correlation with TSR (φ = 0.17, *p* = 0.112) and a negative correlation with TILs (φ = -0.19, *p* = 0.085). CLNM also exhibited significantly positive correlations with volumetric metrics across all four habitats (ρ = 0.27, 0.22, 0.31, 0.28; *p* = 0.013, 0.040, 0.005, 0.009, respectively), as well as tumoral size-related metrics MD and DOI (ρ = 0.34, 0.36; *p* = 0.002, 0.001, respectively).

## Discussion

This study proposed an IVIM-DWI-based habitat imaging strategy for the noninvasive, preoperative assessment of key histopathological features, namely TSR and TILs, in patients with OSCC. We demonstrated that distinct intratumoral subregions defined by characteristic combinations of *D*_*t*_ and *f* values were significantly associated with TSR and TIL levels. These findings suggested that IVIM-DWI-derived habitat metrics may capture biologically relevant microstructural patterns underlying tumor-stroma composition and immune infiltration, supporting their potential role as imaging surrogates for biological stratification and treatment guidance in OSCC.

Habitat imaging offers a voxel-wise, multiparametric framework for characterizing intratumoral heterogeneity [24]. In this study, four IVIM-DWI-derived habitats were defined based on combinations of *D*_*t*_ and *f*, representing putative diffusion–perfusion phenotypes within the tumor microenvironment. Importantly, TSR, TILs, and CLNM were assessed as whole-tumor pathological phenotypes, reflecting stromal abundance, immune activity, and metastatic status, respectively. In contrast, habitat imaging was used to describe spatially heterogeneous intratumoral microstructural patterns at the voxel level. Rather than attempting direct spatial correspondence between imaging habitats and histopathological regions, we focused on evaluating whether specific imaging-defined subregional patterns were systematically associated with these global pathological states.

Our findings indicated that tumors with high TSR exhibited significantly elevated *D*_*t*_ values in hypocellular habitats (subregions 3 and 4), while neither the relative spatial proportions nor volumes of these habitats independently predicted TSR. This pattern suggested that TSR-related differences are more likely driven by microstructural remodeling within pre-existing habitats. High TSR, reflecting stroma-rich tumors, has been consistently linked to unfavorable prognosis and is commonly attributed to desmoplastic reactions, activation of cancer-associated fibroblasts, and immune-excluded microenvironments [23]. Prior studies of desmoplastic and fibrotic tumors have demonstrated that stromal collagen deposition expands the extracellular matrix and increases diffusion metrics such as *D*_*t*_ or ADC, reflecting reduced cellular packing alongside enlarged extracellular spacing [25, 26]. Consistent with this biophysical framework, the elevated *D*_*t*_ value observed in high-TSR tumors may represent stromal matrix expansion occurring within existing imaging-defined habitats rather than preferential growth of specific compartments. Such stromal remodeling has been proposed to form a physical and biochemical barrier to immune cell infiltration; however, the present study was not designed for spatial histologic validation, and this interpretation should therefore be considered hypothesis-generating.

Conversely, tumors with high TILs demonstrated a pattern more consistent with habitat redistribution than with isolated diffusion changes. Specifically, high-TIL tumors exhibited a higher proportion of the cellular-dominant, high-perfusion habitat (subregion 2), accompanied by a relative reduction in stromal-dominant habitat (subregion 3). This compositional redistribution suggested a shift toward regions characterized by higher cellular density and perfusion-related features. Notably, subregion 2 also demonstrated lower mean *D*_*t*_ values in high-TIL tumors, indicating reduced water mobility that may reflect increased cellular packing within these regions. A plausible interpretation is that immune cell infiltration contributes to localized microstructural crowding while remaining compatible with microvascular functionality. This interpretation is consistent with prior observations that vessel density tends to correlate positively with TIL abundance, whereas fibrotic stromal expansion is frequently associated with diminished lymphocytic infiltration [27–30].

Unlike the selective habitat alterations observed for TSR and TILs, CLNM positivity was characterized by volumetric expansion across multiple habitats, suggesting a dominant influence of overall tumor growth rather than targeted microenvironmental remodeling. This pan-habitat enlargement parallels the observation that CLNM-positive tumors tended to exhibit greater tumor size and DOI, indicating that tumor burden may partially contribute to the observed habitat enlargement. Previous studies have shown that stroma-rich OSCC is associated with invasive behavior, poor prognosis, and an increased likelihood of nodal metastasis [3, 30]. While a strong correlation exists between stroma-dominant habitat expansion and CLNM, it remains to be determined whether this association is driven primarily by tumor size or by metrics that are inherently indicative of biological aggressiveness. In our analysis, although the discriminative performances of the clinical-only and habitat-only models were comparable, integrating habitat features with clinical parameters yielded a modest yet consistent improvement in predictive performance. This finding suggests that habitat metrics may provide complementary biological information beyond conventional markers of tumor burden, supporting their potential value in risk stratification.

The observed performance advantage of habitat-based models over conventional whole-tumor diffusion metrics underscored the potential value of spatially resolved imaging analysis. While global ADC or mean *D*_*t*_ values represented averaged diffusion behavior, habitat imaging preserved information on intratumoral heterogeneity and allowed differentiation of diffusion–perfusion patterns relevant to stromal and immune phenotypes. Nevertheless, these findings should be interpreted within the context of an exploratory imaging-pathology association study.

IVIM-DWI-based habitat imaging holds significant translational potential. Unlike contrast-based techniques, it offers a noninvasive and contrast-free modality that is suitable for longitudinal monitoring. By capturing both diffusion and perfusion characteristics, habitat-derived imaging metrics may provide complementary preoperative information on stromal and immune phenotypes, thereby enabling a more comprehensive characterization of the tumor microenvironment. Such information may ultimately support individualized clinical decision-making, including patient stratification, treatment planning, and therapeutic response monitoring.

Several limitations should be acknowledged. First, the sample size was modest, and external validation in independent cohorts is required. Second, TSR and TILs were analyzed as global pathological phenotypes derived from representative histological sections, whereas IVIM-DWI–based habitat imaging captures voxel-wise intratumoral heterogeneity across the entire tumor. The absence of spatially matched radiologic-pathologic correspondence precluded direct subregional validation in the present study. Future investigations integrating whole-slide digital pathology, spatially resolved immunohistochemical mapping, and three-dimensional image-pathology co-registration may enable more precise validation of the biological underpinnings of individual imaging-defined habitats.

In conclusion, these findings collectively demonstrate that habitat-based imaging provides a voxel-wise, multiparametric framework for characterizing heterogeneous intratumoral microstructural patterns underlying tumor-stroma composition and immune infiltration. This noninvasive approach may support preoperative risk stratification and facilitate personalized treatment planning in OSCC.

## Supplementary information

**Additional file 1:** **Table S1.** Comparison of clinical and pathological characteristics between CLNM-negative and CLNM-positive groups. **Table S2.** Inter-observer reproducibility for independent IVIM-DWI-derived habitat metrics. **Table S3.** Predictive performance of univariates and models for CLNM. **Table S4.** Mean AUC from five-fold cross-validation for predicting TSR and TILs levels. **Fig. S1.** ROC Curves of five-fold cross-validation for Predicting (a) TSR and (b) TILs Levels.

## Data Availability

The datasets analyzed in the current study are not publicly available due to ethical principles. However, the data are available from the corresponding author upon reasonable request.

## Abbreviations

ADC: Apparent diffusion coefficient
AUC: Area under the receiver operating characteristic curve
CE-T1WI: Contrast-enhanced T1-weighted imaging
CI: Confidence interval
CLNM: Cervical lymph node metastasis
DOI: Depth of invasion
ICC: Intraclass correlation coefficient
IVIM-DWI: Intravoxel incoherent motion diffusion-weighted imaging
MD: Maximal diameter
MRI: Magnetic resonance imaging
OR: Odds ratio
OSCC: Oral squamous cell carcinoma
ROC: Receiver operating characteristic
ROI: Region of interest
T1WI: T1-weighted imaging
T2WI: T2-weighted imaging
TILs: Tumor-infiltrating lymphocytes
TME: Tumor microenvironment
TSR: Tumor-stroma ratio
