# Supplementary material for: Imaging tumor microenvironment heterogeneity in oral squamous cell carcinoma using IVIM DWI habitat analysis
Source: Eur Radiol Exp. 2026 Sep 9;10:124. doi: 10.1186/s41747-026-00794-z (PMC13558524; doi:10.1186/s41747-026-00794-z)
Supplement: Supplementary file 1 [file 41747_2026_794_MOESM1_ESM.pdf]

Imaging tumor microenvironment heterogeneity in oral squamous cell carcinoma using IVIM DWI habitat analysis

ELECTRONIC SUPPLEMENTARY MATERIAL

Eur Radiol Exp (2026) Li S, Zheng X, Huang Y, Ren J, Wu Y

Table S1. Comparison of clinical and pathological characteristics between CLNM-negative and CLNM-positive groups.

	CLNM-negative (n=39)	CLNM-positive (n=45)	*p*-value
Age (years)	57.7 ± 16.6	55.1 ± 15.2	0.454
Sex (*n*, %)			0.783
Male	24 (61.5)	29 (64.4)	
Female	15 (38.5)	16 (35.6)	
Subsite (*n*, %) (*n*, %)			0.383
Tongue	34 (87.2)	41 (91.1)	
Oral floor	2 (5.1)	1 (2.2)	
Cheek	0 (0.0)	2 (4.4)	
Gingiva	3 (7.7)	1 (2.2)	
cT Stage (*n*, %)			**0.011**
T1-2	20 (51.3)	11 (24.4)	
T3-4	19 (48.7)	34 (75.6)	
TSR (*n*, %)			0.109
Low-TSR	25 (64.1)	21 (46.7)	
High-TSR	14 (35.9)	24 (53.3)	
TILs (*n*, %)			0.083
Low-TILs	16 (41.0)	27 (60.0)	
High-TILs	23 (59.0)	18 (40.0)	
MD (cm)	2.5 ± 0.9	3.3 ± 1.3	**0.002**
DOI (cm)	1.1 ± 0.5	1.7 ± 1.0	**< 0.001**

Note: Data are expressed as mean ± standard deviation or number (percentage). Bold denotes statistically significant results ($p < 0.05$).

CLNM, cervical lymph node metastasis; cT stage, clinical T stage; DOI, depth of invasion; MD, maximal diameter; OSCC, oral squamous cell carcinoma; TSR, tumor-stroma ratio; TILs, tumor-infiltrating lymphocytes.

Table S2. Inter-observer reproducibility for independent IVIM-DWI-derived habitat metrics.

**Metrics**	**ICC (95% CI)**
Subregion3_Mean_D_t	0.875 (0.826, 0.924)
Subregion4_Mean_D_t	0.895 (0.854, 0.936)
Subregion2_Mean_D_t	0.914 (0.880, 0.947)
Subregion3_Percentage	0.978 (0.970, 0.987)

Note: CI, confidential interval; ICC, intraclass correlation coefficient; IVIM-DWI, intravoxel incoherent motion-diffusion weighted imaging.

Table S3. Predictive performance of univariates and models for CLNM.

	AUC (95% CI)	Sensitivity (95% CI)	Specificity (95% CI)	Accuracy (95% CI)
Clinical baseline model	0.702 (0.592, 0813)	0.622 (0.465, 0.762)	0.744 (0.579, 0.870)	0.679 (0.568, 0.776)
Habitat metrics model	0.732 (0.626, 0.839)	0.578 (0.422, 0.723)	0.846 (0.695, 0.941)	0.702 (0.593, 0.797)
Combined model	0.748 (0.645, 0.851)	0.600 (0.443, 0.743)	0.795 (0.635, 0.907)	0.690 (0.580, 0.787)

Note: Clinical baseline model: MD + DOI; Habitat metrics model: Subregion 1_volume + Subregion 3_volume + Subregion 4_volume; Combined model: clinical baseline model + habitat metric model.

ADC, apparent diffusion coefficient, AUC, area under the curve; CI, confidential intervals; CLNM, cervical lymph node metastasis.

Table S4. **Mean AUC from five-fold cross-validation for predicting TSR and TILs levels**.

	AUC (95% CI)	Sensitivity (95% CI)	Specificity (95% CI)	Accuracy (95% CI)
**Prediction of TSR level**				
Mean ROC	0.826 (0.644, 1.000)	0.921 (0.832, 1.000)	0.744 (0.565, 0.924)	0.822 (0.697, 0.947)
**Prediction of TILs level**				
Mean ROC	0.777 (0.634, 0.920)	0.800 (0.518, 1.000)	0.608 (0.465, 0.752)	0.704 (0.494, 0.915)

Note: AUC, are under the curve; CI, confidence interval; TILs, tumor-infiltrating lymphocytes; TSR, tumor-stroma ratio; ROC, region of interest.

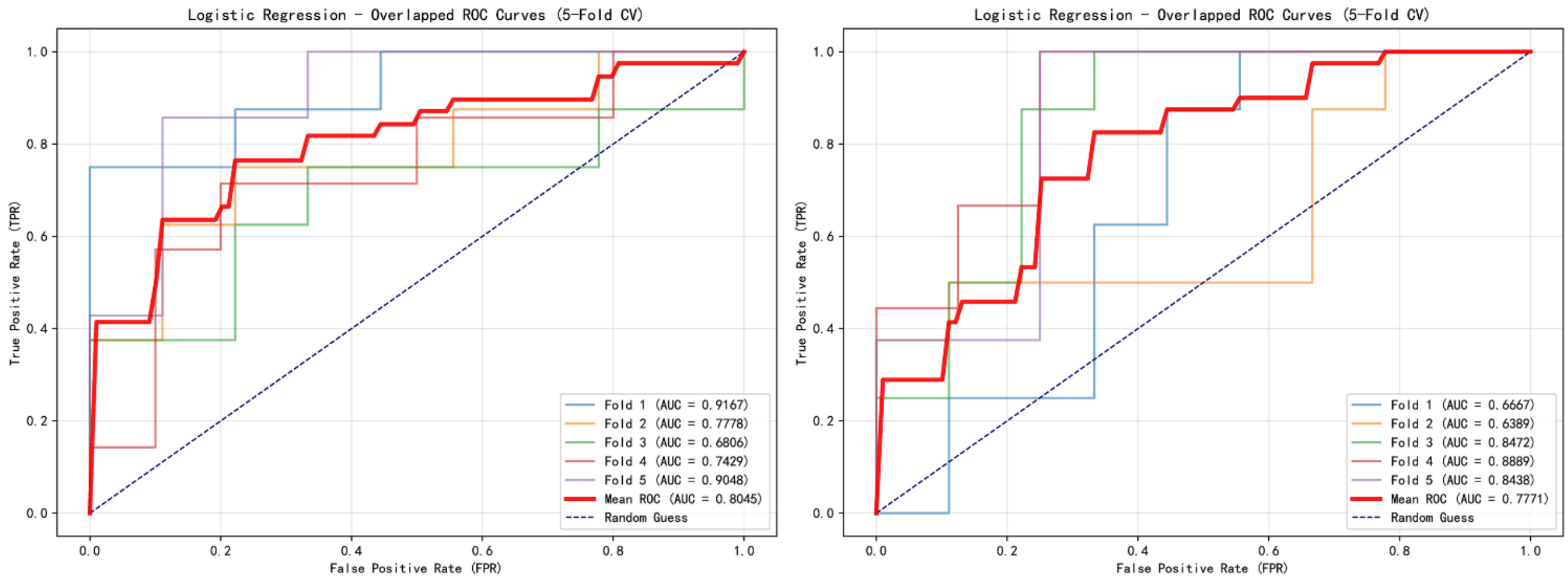

Fig. S1 ROC Curves of five-fold cross-validation for Predicting (a) TSR and (b) TILs Levels.

Note: ROC, receiver operating characteristic; TILs, tumor-infiltrating lymphocytes; TSR, tumor-stroma ratio.
